# The C-8-*S*-isomers of ergot alkaloids — a review of biological and analytical aspects

**DOI:** 10.1007/s12550-023-00507-0

**Published:** 2023-11-13

**Authors:** Jensen E. Cherewyk, Barry R. Blakley, Ahmad N. Al-Dissi

**Affiliations:** 1 Department of Veterinary Biomedical Sciences, Western College of Veterinary Medicine, University of Saskatchewan, Saskatoon, SK S7N 5B4 Canada; 2 Department of Veterinary Pathology, Western College of Veterinary Medicine, University of Saskatchewan, Saskatoon, SK S7N 5B4 Canada

**Keywords:** Contamination, Bioactivity, Analytical, Regulations, *S*-epimers

## Abstract

Ergot alkaloids are secondary metabolites that are produced by fungi and contaminate cereal crops and grasses. The ergot alkaloids produced by *Claviceps purpurea* are the most abundant worldwide. The metabolites exist in two configurations, the C-8-*R*-isomer (*R*-epimer) and the C-8-*S*-isomer (*S*-epimer). These two configurations can interconvert to one another. Ergot alkaloids cause toxic effects after consumption of ergot-contaminated food and feed at various concentrations. For bioactivity reasons, the C-8-*R*-isomers have been studied to a greater extent than the C-8-*S*-isomer since the C-8-*S*-isomers were considered biologically inactive. However, recent studies suggest the contrary. Analytical assessment of ergot alkaloids now includes the C-8-*S*-isomers and high concentrations of specific C-8-*S*-isomers have been identified. The inclusion of the C-8-*S*-isomer in regulatory standards is reviewed. This review has identified that further research into the C-8-*S*-isomers of ergot alkaloids is warranted. In addition, the inclusion of the C-8-*S*-isomers into regulatory recommendations worldwide for food and feed should be implemented. The objectives of this review are to provide an overview of historic and current studies that have assessed the C-8-*S*-isomers. Specifically, this review will compare the C-8-*R*-isomers to the C-8-*S*-isomers with an emphasis on the biological activity and analytical assessment.

## Introduction

Cereal and grass crops can be infected by fungi which are associated with ergot. The fungi include *Claviceps*, *Penicillium*, *Aspergillus*, and *Epichloë coenophiala*, also known as *Acremonium coenophialum* or *Neotyphodium coenophodium* (Blaney et al. 2009; Krska and Crews 2008; Scott 2009; Gerhards et al. 2014; Chen et al. 2017)*.* One of the most notable fungal infections is *Claviceps purpurea* from the *Claviceps* genus. The ascospores from the fungus *Claviceps purpurea* land on the stigmas of crops, initially infecting the ovary of the plant (Miedaner and Geiger 2015). An infected ovary produces a mass called “honeydew,” which can spread to other plants by insects, rain, and equipment. The honeydew hardens into a sclerotia, replacing the seed of the crops. The sclerotia, also known as an ergot body, has a dark outer coating (Menzies & Turkington 2015).

The most susceptible crops to ergot are cross-pollinators such as triticale and rye with a longer flowering stage (Menzies and Turkington 2015). Wheat, barley, oats, and millet can also become infected (Agriopoulou 2021). An infected wheat crop may have higher concentrations of ergot than a rye crop in certain geographic locations (Schummer et al. 2018). Grasses can also become infected (Arroyo-Manzanares et al. 2017; Klotz et al. 2018). Cool and wet environmental conditions promote ergot infection in crops and grasses (Agriopoulou 2021). In certain years, ergot infections in crops or grasses may be higher than other years due to favorable environmental conditions.

Humans and animals are exposed to ergot through consumption of ergot-contaminated food and feed products, respectfully. Ergot has had substantial adverse effects on humans and animals. Historically, it was not understood why ergot-contaminated products caused the adverse effects. Today, it is known that the adverse effects are associated with compounds within the ergot bodies known as ergot alkaloids.

Ergot sclerotia contain secondary metabolites, ergot alkaloids, which are a group of nitrogenous organic compounds (Komarova and Tolkachev 2001), concentrated on the outer edge of the sclerotia, with lower concentrations in the interior (Young et al. 1983). Ergot alkaloids are defined as secondary metabolites since they are produced by the fungi but not involved in normal physiological processes of the fungi, as opposed to primary metabolites that contribute to physiological processes such as growth, reproduction, or development (Susan 2023). There are 90 different known ergot alkaloids that have been isolated worldwide (Liu and Jia 2017). A considerable amount of ergot alkaloids is produced within the ergot sclerotia by the *Claviceps* genus (Komarova and Tolkachev 2001). Ergot alkaloids can be divided into major groups including simple lysergic acid derivatives, peptide alkaloids, ergopeptam alkaloids, and clavine alkaloids which are classified based on their chemical structures (Komarova and Tolkachev 2001; Krska and Crews 2008; Strickland et al. 2011; EFSA 2012; Crews 2015; Sharma et al. 2016).

There are six common ergot alkaloids produced by *Claviceps purpura* globally. These alkaloids include ergocristine, alpha and beta-ergocryptine, ergocornine, ergometrine (also referred to as ergonovine), ergosine, and ergotamine (Crews 2015; Chung 2021). The six common ergot alkaloids are classified as peptide alkaloids, apart from ergometrine which is classified as a simple lysergic acid derivative. Another common ergot alkaloid is ergovaline which is produced by *Epichloë coenophiala* (*Neotyphodium* spp.) (Shappell and Smith 2005; Blaney et al. 2009).

**Fig. 1:**
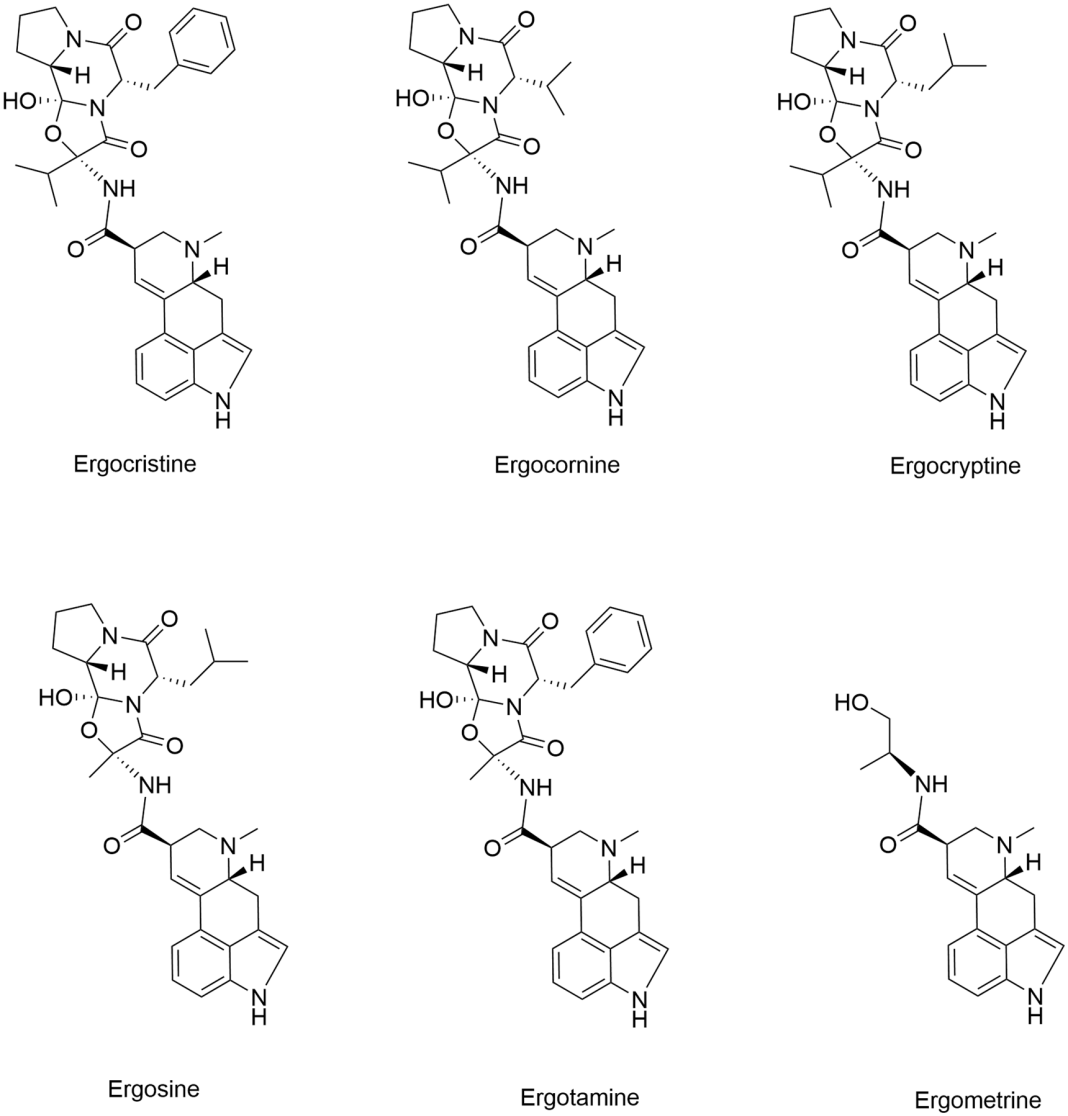
Chemical structures of the six common ergot alkaloids (National Center for Biotechnology Information - PubChem)

All ergot alkaloids share a common chemical structure containing a tetracyclic ergoline ring of lysergic acid (EFSA 2005). Peptide alkaloids have an amino acid ring system attached to the ergoline ring system (Krska and Crews 2008). Side chain variations of the amino acids which constitute the amino acid ring system define the specific ergot alkaloid (Fig. 1). Lysergic acid derivatives do not contain an amino acid ring system. Other derivatives of ergot alkaloids, such as dihydro-derivatives of the peptide alkaloids, can be formed synthetically (dihydroergotamine) or within sclerotia (dihydroergosine) (EFSA 2012). All ergot alkaloids have various configurations which are defined by their chemical structure.

**Fig. 2:**
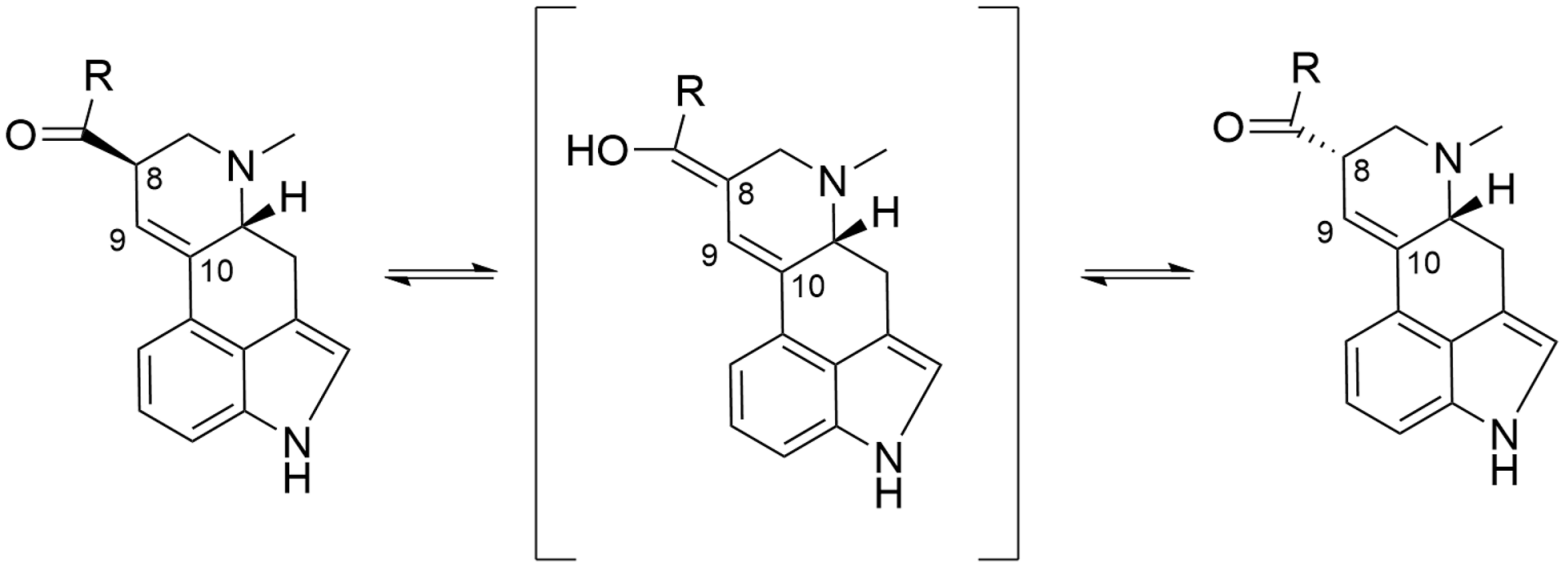
Left: C-8-*R*-isomer (*R*-epimer). Middle: intermediate structure. Right: C-8-*S*-isomer (*S*-epimer) (Cherewyk et al. 2022b)

Ergot alkaloids have multiple chiral centers, and the rotation defines the specific configuration. A left-hand rotation at the carbon 8 chiral center adjacent to the 9–10 double bond forms a C-8-*R*-isomer and a right-hand rotation forms a C-8-*S*-isomer (Komarova and Tolkachev 2001; Krska and Crews 2008) (Fig. 2). However, other terminology states that a clockwise rotation at a chiral center is defined as *R* (rectus) vs *S* (sinister) (Cieplak and Wisniewski 2001). The C-8-*S*-isomer can also be defined with the prefix “iso” (e.g., isolysergic acid), compared to the C-8-*R*-isomer (e.g., lysergic acid) (Jastrzębski et al. 2022). A rotation at the carbon 5 (C5) of an ergot alkaloid may also signify a specific configuration and can be defined by a “d” (e.g., d-lysergic acid) or “l” (e.g., l-lysergic acid). The ergot alkaloids in either the C-8-*R*-isomer or C-8-*S*-isomer configuration are naturally in the d configuration at the carbon 5 (Krska et al. 2008b; Klotz et al. 2010; National Center for Biotechnology Information - PubChem: Ergocristine, Ergosine, Ergocornine, Ergocryptine, Ergotamine, Ergometrine).

In this review, the focus is on the C-8 rotation, and specifically the C-8-*S*-isomer. The C-8-*R*-isomer and C-8-*S*-isomer are referred to as the *R*-epimer and *S*-epimer, respectively. The *R*-epimers are defined with a -ine suffix (e.g., ergotamine) and the *S*-epimers with a -inine suffix (e.g., ergotaminine). The *R* and *S*-epimers are interconvertible (Crews 2015), and pass through an intermediate configuration (Andrae et al. 2014) during the energetic conversion. The rationale for focusing on the *S*-epimers is due to the lack of studies on that configuration, which may be because the *S*-epimers being historically deemed as biologically inactive or the relative abundance of the *S*-epimers were not considered, which is discussed further in the review.

## Biological evaluation of ergot alkaloids (*R* and *S*-epimers)

### Biological activity of the *R* and *S*-epimers

Differences between the *R* and *S*-epimers of ergot alkaloids are reported, specifically in terms of bioactivity. Bioactivity is defined as “any response from or reaction in living tissue” (Mosby’s Medical Dictionary 2017, p. 209). The *R*-epimers are deemed to have biological activity (Komarova and Tolkachev 2001). In contrast, the *S*-epimers are reported to be inactive (Barger and Carr 1907; Berde and Schild 1978; Pierri et al. 1982; Schiff 2006; Krska and Crews 2008; Blaney et al. 2009; Smith et al. 2009; Nichols 2012; Dänicke 2016; Guo et al. 2016; Bryła et al. 2019) or have weak activity (Komarova and Tolkachev 2001; Haarmann et al. 2009; Strickland et al. 2011; Stanford et al. 2018). However, recent studies have demonstrated potential bioactivity of the *S*-epimers of peptide alkaloids (Mulac et al. 2012; Cherewyk et al. 2020; Cherewyk et al. 2022a, b, c).

Many studies that describe inactivity of *S*-epimers reference studies dating to the 1970s or prior (Stadler and Stürmer 1970; Berde and Schild 1978; White 1938a, b). In Berde and Schild (1978), the authors state that the *S*-epimers are less active than the *R*-epimers; however, they do not provide a supporting reference. In a table assessing adrenergic receptor blocking activity, ergotaminine was reported to have no activity when a nictitating membrane of a cat was used (Bacq 1934; Salzmann and Bucher 1978). Berde and Schild (1978) also reported that specific ergot epimers, namely, the diastereomers of ergotamine and dihydroergotamine, do not have bioactivity, where they reference Stadler and Stürmer (1970, 1972). The reference Stadler and Stürmer (1972) could not be found. In Stadler and Stürmer (1970), the non-bioactivity of the diastereomers (isomers) of ergotamine and dihydroergotamine was allegedly summarized from multiple papers and was tested with 11 pharmacological assays, which were not described in the study with suitable references. Another study reported the inactivity of an *S*-epimer, 5S, 8S-(−)-lysergic acid diethylamide (LSD) with 2500-fold lower activity than the corresponding C-8-*R*-isomer (Nichols 2012). However, when assessing the reference of that statement, there was no 5S, 8S-(−)-LSD analyzed (Bennett and Snyder 1976). The only C-8-*S*-isomer analyzed in the reference was d-iso-lysergic acid amide, which is different from LSD. In Bennett and Snyder (1976), d-iso-lysergic acid amide had an inhibitory concentration affecting 50% of the population (IC50) of 100–200 nM compared to d-LSD with IC50s of 8–10 nM, when assessed with radioligand binding assays. Although the IC50s for the *S*-epimer are higher than the *R*-epimer, the *S*-epimer IC50s are still relatively low. The authors further state that l-LSD is inactive; however, l-LSD is not a C-8-*S*-isomer.

Studies referencing the lack of biological activity of the C-8-*S*-isomers appear to have erroneous or incomplete information. Some studies only examined one ergot alkaloid or class of alkaloids or mistake the C-8-*S*-isomer for the C5 isomer (Stadler and Stürmer 1970; Bennett and Snyder 1976; Salzmann and Bucher 1978). The specific alkaloids, dose, endpoints, methods, and animal species used could all contribute to the uncertainty of the C-8-*S*-isomers related to bioactivity (Saamely 1978). Studies today define all C-8-*S*-isomers as non-bioactive or weakly bioactive and reference studies that assessed only one C-8-*S*-isomer with methods that may not be optimal. When examining the historical data on the non-bioactivity of the *S*-epimers, the methods and results are questioned. Limited studies assessing the bioactivity of the *S*-epimers report the ratio of the epimers within the experiments. However, the experimental conditions used encourage the epimerization of the *R*-epimer to the S-epimer, and not vice versa. Conditions such as temperature, solvent, pH, and time were all identified and set to minimize the back epimerization of the *S* to the *R*-epimer. A study assessing ratios of ergot epimers under physiological conditions had demonstrated high concentration stability, therefore minimal epimerization, of a specific *S*-epimer (Mulac et al. 2012). Epimerization of ergot alkaloids is discussed further in the review. An updated assessment of the *S*-epimers of ergot alkaloids to further understand their bioactivity and related mechanisms is warranted.

### The *R* and *S*-epimer — receptor interactions 

**Fig. 3:**
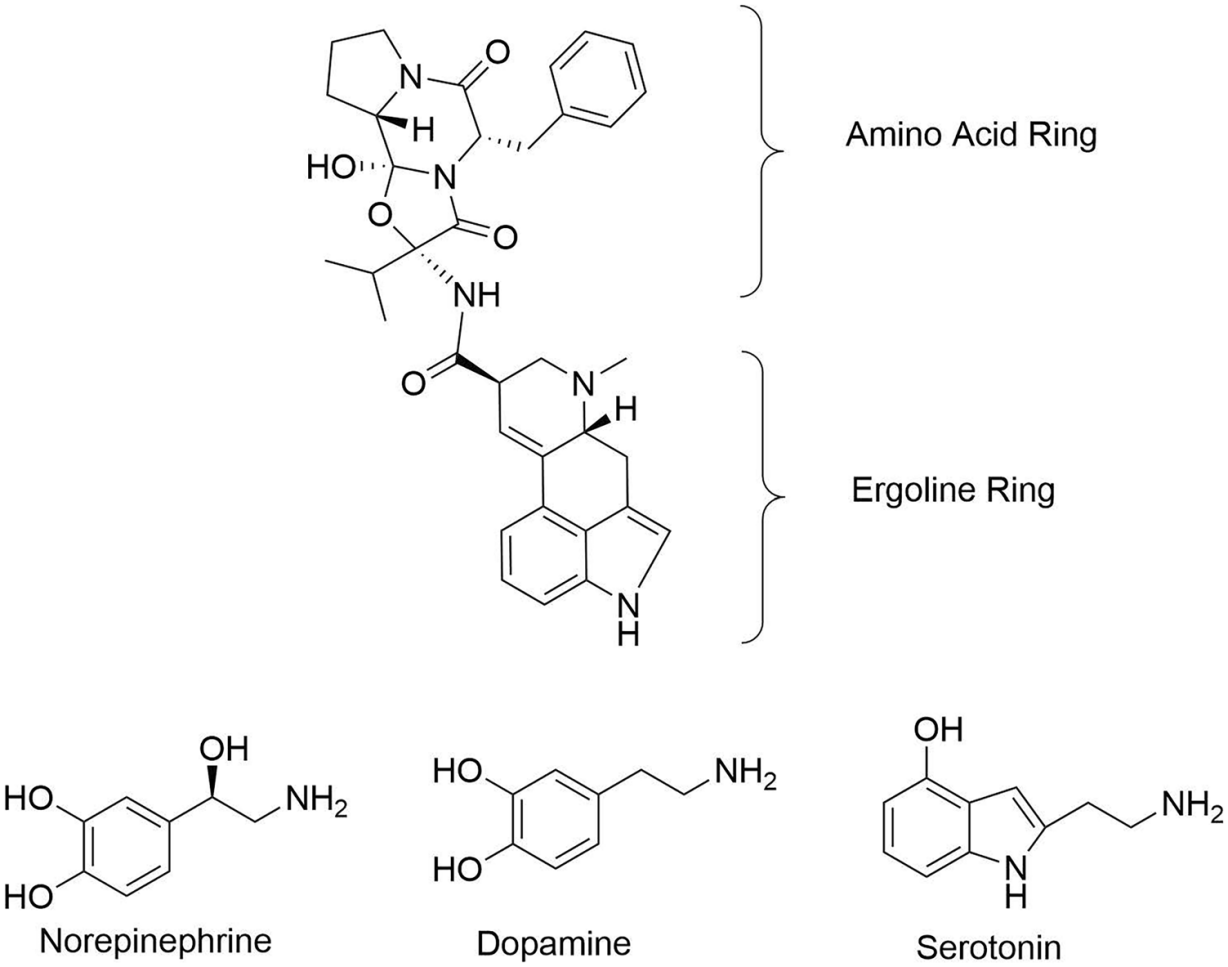
Chemical structures representing the amino acid and ergoline ring system, and the biogenic amines, norepinephrine, dopamine, and serotonin (chemical structures were recreated from PubChem and Wikimedia Commons: Dopamine, Norepinephrine, Serotonin)

The biological activity of ergot alkaloids is related to their interaction with biogenic amine receptors. The ergoline ring system of ergot alkaloids is structurally similar to the biogenic amines norepinephrine, dopamine, and serotonin (5-HT) (Berde 1980; Klotz 2015a) (Fig. 3). The similarity of the structures allows ergot alkaloids to bind to dopamine, adrenergic (also known as adrenoceptors), and 5-HT receptors (Hollingsworth et al. 1988; Klotz 2015b). The ergoline ring of the chemical structure has been defined as the pharmacophore of peptide alkaloids (Weber 1980; Reddy et al. 2020). The different classes of ergot alkaloids and differing amino acid ring side chains for the peptide alkaloids result in varying affinities to the receptors and subsequent responses (Strickland et al. 2011; Klotz 2015b). Structural differences of ergot alkaloids may influence the signal transduction after binding to a receptor (Klotz et al. 2010). Ergot alkaloids, specifically the *R*-epimers, interact with the biogenic receptors in multiple ways.

Ergot alkaloids have been defined as agonists, partial agonists, and antagonists to dopamine, alpha adrenergic, and 5-HT receptors (Hollingsworth et al. 1988; Pertz and Eich 1999; Klotz et al. 2016). The binding of ergot alkaloids to various subtypes of each receptor class has been investigated. The 5-HT 1 (MacLennan and Martin 1990; Schöning et al. 2001; Schiff 2006), 5-HT 2 (Dyer 1993; Oliver et al. 1993; Schöning et al. 2001; Schiff 2006; Görnemann et al. 2008; Klotz 2015b; Klotz et al. 2016, 2018), 5-HT 2A, 5-HT 2B (Klotz 2015b), alpha adrenergic 1 (Oliver et al. 1993; Schöning et al. 2001; Görnemann et al. 2008), alpha 2 adrenergic receptors (Roquebert et al. 1984; Oliver et al. 1998; Görnemann et al. 2008; Yonpiam et al. 2021; Klotz et al. 2016), dopamine 1 (Saper and Silberstein 2006), and dopamine 2 receptors (Pertz and Eich 1999; Görnemann et al. 2008) have all demonstrated to interact with ergot alkaloids upon exposure. Factors that influence the involvement of receptors include the specific ergot alkaloid, animal species, tissue type, dose, and experimental conditions assessed (Schiff 2006; Klotz 2015b). For example, the 5-HT 2A receptor was the primary serotonin receptor causing effects following ergot alkaloid exposure in the vasculature (Klotz et al. 2018; Trotta et al. 2018). However, the receptors involved with ergot may depend on the type of vasculature assessed. Alpha adrenergic receptors are more predominant in peripheral vasculature, and specific receptors may not be present in all blood vessels (Klotz et al. 2018; Liu et al. 2020). To assess specific receptors in vasculature that are involved with ergot alkaloid exposure, a combination of the ergot alkaloid and agonists/antagonists of the receptors have been utilized (Dyer 1993; Schöning, et al. 2001; Klotz et al. 2016). Table 1 summarizes studies that have reported the involvement of a specific receptor after ergot alkaloid exposure and includes the specific ergot alkaloid(s) used in the study. Most studies assessing the involvement and interaction of receptors following ergot alkaloid exposure have only focused on the *R*-epimers.

**Table 1:** A summary of studies reporting the involvement of specific receptors following ergot alkaloid exposure and which *R*-epimer, from the six common ergot alkaloids and ergovaline, was reported along with the relationship observed

**Receptor**	**Ergot alkaloid**	**Agonist/antagonist**	**Reference**
5-HT_1_	Ergometrine	Agonist	MacLennan and Martin (1990)
5-HT_1A_	Ergometrine Ergotamine	Agonist	Pertz and Eich (1999)
5-HT_1B/D_	Ergometrine Ergovaline Ergotamine	Agonist	Pertz and Eich (1999), Schöning et al. (2001), Schiff (2006) and Klotz et al. (2018)
5-HT_2_	Ergometrine Ergovaline	Agonist	MacLennan and Martin (1990) and Dyer (1993)
5-HT_2A_	Ergometrine Ergotamine Ergovaline	Agonist Antagonist	Hollingsworth et al. (1988), Pertz and Eich (1999), Klotz et al. (2016), Schöning et al. (2001) and Klotz et al. (2018)
5-HT_2B_	Ergotamine	Antagonist	Pertz and Eich (1999)
5-HT_2C_	Ergotamine	Antagonist	Pertz and Eich (1999)
5-HT_5A/5B_	Ergotamine	n/a	Pertz and Eich (1999) and Tfelt-Hansen et al. (2000)
5-HT_4_	Ergotamine	Agonist	Jacob et al. (2023)
Alpha-adrenergic	Ergotamine	Partial agonist	Schiff (2006)
Alpha-2	Mixture	Agonist	Oliver et al. (1993) and Tfelt-Hansen et al. (2000)
Alpha-2a	Ergovaline	Antagonist	Klotz et al. (2016)
Alpha-2c	Ergovaline	Antagonist	Klotz et al. (2016)
Alpha-1	Ergovaline Mixture	Partial agonist	Tfelt-Hansen et al. (2000), Schöning et al. (2001) and Yonpiam et al. (2021)
Alpha-1a	Ergotamine Ergocristine	Partial agonist Antagonist	Görnemann et al. (2008)
Alpha-1b	Ergotamine Ergocristine	Partial agonist Antagonist	Görnemann et al. (2008)
Alpha-1d	Ergotamine Ergocristine	Partial agonist Antagonist	Görnemann et al. (2008)
D_2_	Ergotamine	Agonist	Tfelt-Hansen et al. (2000)

The *R*-epimers of ergot alkaloids are known to have high affinity to the serotonin, alpha adrenergic, and dopamine receptors (Haarmann et al. 2009; Ivanova and Spiteller 2012). The *R*-epimers have a higher affinity to alpha 2 adrenergic receptors compared to the alpha 1 adrenergic receptors (Klotz et al. 2016). The high affinity may be due to the slow dissociation and association of the *R*-epimers to the receptors (Schöning et al. 2001; Unett et al. 2013). Irreversible binding of the *R*-epimers to receptors has also been speculated (Schöning et al. 2001). In addition, the *R*-epimers have time-dependent binding affinities with differing *K*_i_ values at 5 min vs 5 h post-exposure (Unett et al. 2013). These studies demonstrate the involvement of the *R*-epimers of ergot alkaloids in receptor binding and activation. However, the involvement of the *S*-epimers in receptor binding and comparison of the data to the *R*-epimers is limited.

The *S*-epimers were reported to have weak activity in terms of receptor activation (Mulac and Humpf 2011; Mulac et al. 2012, 2013). However, *S*-epimers, 8S-lisuride and terguride, demonstrated agonist and antagonist properties to histamine and 5-HT receptors, respectively (Pertz et al. 2006; Kekewska et al. 2012), using receptor and ex vivo assays. The 8S-lisuride demonstrated greater potency than its corresponding C-8-*R*-isomer. The 8S-lisuride and terguride are ergot alkaloid derivatives that have similar ergoline ring chemical structures to ergot alkaloids. Another *S*-epimer, 8-alpha-ergoline, demonstrated relatively high affinity with *pK*_i_ values of 6.92–8.52 to alpha 1 and alpha 2 adrenergic receptors, using a radioligand binding assay (Okumura et al. 1988). In addition, a recent radioligand study demonstrated that isolysergol, a C-8-*S*-isomer, binds with high potency to four 5-HT receptor subtypes and had similar potency to the C-8-*R*-isomer (Tasker and Wipf 2022). Each compound, the C-8-*S*-isomer and C-8-*R*-isomer, had a d configuration at the C5 position to demonstrate sufficient potency. Receptor binding of these *S*-epimers encourages further studies to assess the involvement of other classes of *S*-epimers in receptor binding. The *R* and *S*-epimer of a novel ergopeptine alkaloid had only slight differences in affinity to the adenosine and dopamine receptors (Vendrell et al. 2007). Therefore, the authors made a statement, “Concerning the effect of ergolene system chirality, the C8 configuration is not relevant in ergopeptides pharmacology, as the diastereoisomers….showed similar behavior in the four receptors evaluated” (Vendrell et al. 2007, p. 3066). For peptide alkaloids, in silico methods have been used to assess the *S*-epimer-receptor binding relationship and have demonstrated affinity and strong molecular interactions (Dellafiora et al. 2015; Spaggiari et al. 2021; Cherewyk et al. 2023a, b). Further assessments of the *S*-epimer-receptor relationship may lead to further understanding of the biological activity and associated mechanisms.

### Mechanisms of toxic effects of the *R* and *S*-epimers

Ergot alkaloid binding to receptors is the first step of the biological process that leads to downstream apical effects. Serotonin and alpha-adrenergic receptors are located in the smooth muscle cells of vasculature and the interaction between ergot alkaloids and the biogenic receptors negatively affects the vasculature. The most notable biological response is the contraction of various blood vessels in the peripheral and central vasculature (MacLennan and Martin 1990; Oliver et al. 1993; Klotz et al. 2019). Low concentrations of ergot have produced arterial effects (Cowan et al. 2018). Vasculature that have been reported to be affected by *R*-epimers include, but not limited to, the dorsal pedal (metatarsal) artery and vein, mesenteric artery and vein, lateral saphenous vein, ruminal artery and vein, medial palmar artery and vein, and umbilical vasculature (Solomons et al. 1989; Klotz et al. 2006, 2007, 2009, 2010, 2018, 2019; Foote et al. 2012; Egert et al. 2014; Klotz and McDowell 2017; Trotta et al. 2018; Yonpiam 2018).

The *R*-epimers of peptide alkaloids have demonstrated a concentration-dependent response of various vasculature (Klotz et al. 2010; Foote et al. 2011). In addition, a prolonged/sustained vascular contractile response following *R*-epimer exposure has also been observed (Solomons et al. 1989; Klotz et al. 2007; Pesqueira et al. 2014; Klotz 2015b). The rationale for the sustained vascular contractile response is bioaccumulation of ergot alkaloids in the tissues (Klotz et al. 2007, 2009, 2016; Klotz 2015b) or high irreversible affinity binding to receptors (Pesqueira et al. 2014), resulting in persistent receptor signaling and delayed recovery (Klotz et al. 2016). The delayed recovery of vasculature may lead to further adverse implications and potentially reduced elimination of ergot alkaloids. Ergot alkaloid concentrations in vascular tissue have been observed analytically (Klotz et al. 2009). In contrast, lysergic acid amides do not cause a sustained vascular constriction (Berde and Schild 1978; Klotz et al. 2006; Pesqueira et al. 2014). As a result, the lysergic acid amides may not bioaccumulate in vascular tissue; therefore, do not have prolonged effects compared to peptide alkaloids. The effects of ergot alkaloids on the vasculature have been attributed to the *R*-epimers. The contribution of the *S*-epimers to vasculature contraction has remained limited.

The *S*-epimers of peptide alkaloids have caused contractions of an artery and uterus. Ergocristinine, ergocryptinine, ergocornine, and ergotaminine produced a cumulative dose response using a dorsal metatarsal artery (Cherewyk et al. 2020). In addition, ergocristinine demonstrated sustained vascular contraction and appeared to have a slower onset of action compared to the corresponding epimer (Cherewyk et al. 2022c). Likewise, utilizing a rabbit uterus, ergosinine demonstrated similar effects to ergosine, but had slightly weaker and slower onset of action (Saamely 1978). Ergosinine also elicited a contractile response in a guinea pig uterus when assessed with a myocardiograph (White 1938b). Ergometrinine demonstrated 3.9% of uterine contraction compared to ergometrine; however, the concentration used was not provided (Saamely 1978). The authors assessed the uterine contraction at 2–5 min. Potentially, the time at which the contraction was assessed may have been insufficient to observe the response related to the *S*-epimer, especially if the *S*-epimer has a slower onset of action. The observations of the *S*-epimers to cause contraction as observed in the above studies suggest potential bioactivity of the *S*-epimers.

Other studies demonstrating the potential effects of *S*-epimers involve cellular toxicity. The *S*-epimers accumulate in cells (Shappell and Smith 2005; Mulac and Humpf 2011; Mulac et al. 2013). Ergocristinine accumulated to a greater extent than ergocristine in two different cancer cell lines (human colon (HepG2) and human liver (HT-29)) (Mulac et al. 2013). Uptake and accumulation into cells may result in cytotoxicity attributed to the *S*-epimers (Mulac and Humpf 2011). In addition, ergocristinine accumulated in the blood–brain barrier, affecting the integrity of the barrier (Mulac et al. 2012), which could be a potential toxicological mechanism. Ergovalinine crossed intestinal cells at similar rates to ergovaline (Shappell and Smith 2005); therefore, both epimers may contribute to toxic effects following ergot exposure. In addition, ergometrinine demonstrated toxicity in animal smooth muscle cells, supporting that *S*-epimers may contribute to cytotoxicity (Zhang et al. 2014). In a study assessing cAMP in cell lines with adenosine and dopamine receptors, the levels of cAMP did not differ between a specific *R* and *S*-epimer (Vendrell et al. 2007). The potential for *S*-epimers of ergot alkaloids to accumulate, demonstrate toxicity, and produce cAMP in cells encourages further research to understand the bioactivity and mechanisms of *S*-epimers and their potential to cause toxic manifestations.

### Toxic manifestations of the *R *and *S*-epimers

Ergot alkaloids can be consumed orally through contaminated food and feed. Absorption of ergot alkaloids occurs in the gastrointestinal tract, specifically the small intestine (Strickland et al. 2011). The extent of the absorption of the ergot alkaloids is dependent on the chemical structure of each compound (Völkel et al. 2011). Another potential route of absorption is through the lymphatic system (Klotz 2015b). In ruminants, ergot alkaloids can be absorbed during ruminal passage (Völkel et al. 2011). Ergot alkaloids exhibit hepatic metabolism and enterohepatic recirculation (Völkel et al. 2011; Sharma et al. 2016), which may result in low bioavailability. However, if hepatic clearance reaches capacity, the ergot alkaloids may not be metabolized and enter the systemic circulation. The absorption of compounds from the gastrointestinal circulatory system directly to the systemic circulation has also been suggested (Talevi and Bellera 2021). Ergot alkaloids have been documented to accumulate in cells (Mulac and Humpf 2011); therefore, the exposure of low concentrations of ergot alkaloids may still result in adverse effects. Ergot alkaloids are excreted mostly through the urinary and biliary systems (Klotz 2015b). The ergot alkaloid concentrations eliminated do not always equal the concentrations consumed. The potential rationales as to why the concentration consumed does not equal the concentration excreted may be due to accumulation or metabolism of the ergot alkaloids. Accumulation of ergot alkaloids within humans and animals may lead to prolonged adverse effects leading to toxic manifestations.

One of the predominant syndromes of ergot intoxication is gangrenous ergotism (Schiff 2006; Klotz 2015a). Gangrenous ergotism is related to the vascular constriction induced by the *R*-epimers of ergot alkaloids. The constriction of blood vessels leads to reduced blood flow and potentially loss of limbs or extremities such as ears and tail tips (Rahimabadi et al. 2022). Peripheral arteries may be more sensitive to ergot than central arteries (Cowan et al. 2018). Blood flow restriction can also cause harm to reproductive organs (Poole et al. 2018; Klotz et al. 2019; Poole and Poole 2019). Implications of impaired cardiovascular function include altered nutrient transport, temperature regulation, and waste elimination (Strickland et al. 2011). The adverse effects attributed to ergot may lead to health complications in humans and animals, which in severe cases can result in death. Based on the literature, vasoconstriction leading to adverse health effects is a main issue associated with ergot consumption, especially in livestock, and should be a primary focus of studies assessing ergot.

Other manifestations of ergot alkaloid intoxication contributing to the adverse health effects have been reported. Nervous ergotism, which involves the nervous system, is manifested as paranoia, hallucinations, twitches, and spasms (Schiff 2006; Haarmann et al. 2009; Klotz 2015a). Decreased milk production has been observed and is associated with decreased prolactin (Bernard et al. 1993; Klotz 2015a; Burnett et al. 2018; Chohan et al. 2021; Cowan et al. 2023). In addition, decreased weight gain and feed intake (Coufal-Majewski et al. 2017; Dänicke 2015; Klotz 2015a), reduced circulating serotonin (Valente et al. 2020), liver and intestine lesions (Maruo et al. 2018), and altered total bilirubin and albumin concentrations (Dänicke 2016) have been described following ergot alkaloid exposure. The manifestations of ergot exposure cause various health issues, leading to a decrease in the well-being of animals and economic losses. The toxic manifestations following ergot alkaloid exposure have been attributed to the *R*-epimer and not the *S*-epimer. Only historical studies have assessed toxic manifestations following *S*-epimer exposure.

Various *S*-epimers of ergot alkaloids have produced toxic manifestations in animals. Isoergine, an *S*-epimer, produced visual adverse manifestations in monkey, cat, and fowl species (White 1938a). Allegedly, the animals recovered hours later; however, the recovery was based on the observed clinical signs of the organism. Ergosinine produced adverse effects in monkey, cat, rabbit, guinea pig, and fowl species. Slower onset and progress toward recovery was observed compared to ergosine (White 1938a). White (1938a) noted that “if ergosinine were allowed to act for longer than the prescribed time its sympatholytic activity was more marked” (p. 143). Some claims regarding the non-activity of *S*-epimers were based on rectal temperature of mice (White 1938a). The conclusion of these historical studies may be inconsistent with regards to recent studies referencing the non-bioactivity of the *S*-epimers. The lack of current studies addressing the *S*-epimers, and the potential bioactivity of the *S*-epimers, reinforces the need for investigation into the *S*-epimer of ergot alkaloids to further understand their biological relevance. The assessment of the concentrations of both *R* and *S*-epimers of ergot alkaloids in contaminated matrices is important from a food and feed safety perspective, for the protection of human and animal health.

## Analytical evaluation of ergot alkaloids (*R* and *S*-epimers)

### Methods of *R* and *S*-epimer detection and quantification

The toxic nature of ergot alkaloids encourages continuous monitoring and quantifying in food and feed (Drakopoulos et al. 2021). The European Union reported that the six common *R* and *S*-epimers should be monitored (Chung 2021). The quantification of ergot alkaloids has been conducted in multiple food matrices (Liang et al. 2022). For ergot alkaloid analysis, certain variables related to sample handing are important. These include homogeneity, particle size, and amount of sample (European Commission 2013; Chung 2021). Appropriate methods and analysis for both configurations of ergot alkaloids are needed.

There are multiple methods for detection and quantification of ergot alkaloids. The methods include colorimetric analysis, thin layer chromatography, enzyme-linked immunosorbent assay (ELISA), liquid chromatography with ultra-violet (UV) detection or mass spectrometry (MS) detection, and near-infrared spectroscopy (Scott 2007). However, colorimetric, ELISA, and the use of MS without liquid chromatography (LC) separation cannot quantify the *S*-epimers of ergot alkaloids (Scott 2007). Liquid chromatography-florescence detection (Holderied et al. 2019), capillary electrophoresis (Frach and Blaschke 1998), liquid chromatography mass spectrometry (Krska et al. 2008b), and ion mobility mass spectrometry (Carbonell-Rozas et al. 2022) can quantify both *R* and *S*-epimers of ergot alkaloids. Liquid chromatography coupled with tandem mass spectrometry (LC-MS/MS) has been labeled the gold standard for ergot quantification (Tittlemier et al. 2020).

A review of published methods for epimer specific quantification has been prepared recently (Chung 2021). Multiple studies have quantified the six common *R* and six common *S*-epimers (Diana Di Mavungu et al. 2012; Krska et al. 2008b; Malysheva et al. 2013; Guo et al. 2016; Arroyo-Manzanares et al. 2018; Schummer et al. 2018; Holderied et al. 2019; Arroyo-Manzanares et al. 2021; Mulder et al. 2015; Carbonell-Rozas et al. 2021; Poapolathep et al. 2021; Cherewyk et al. 2022a). However, some studies quantified only the *R*-epimer (Lehner et al. 2005; Mohamed et al. 2006; Ruhland and Tischler 2008; Martos et al. 2010; Kowalczyk et al. 2016; Grusie et al. 2017; McKinnon et al. 2018; Shi et al. 2019). Cherewyk et al. (2022a) demonstrated that differences occurred between the *R* and *S*-epimer validation parameters, potentially due to greater ionization of the *S*-epimers than the *R*-epimers. Studies should quantify both configurations associated with the potential epimerization between the *R* and *S*-epimer and the questionable *S*-epimer biological activity. Accurate and reliable assessments for the concentrations of *R* and *S*-epimers of ergot alkaloids are needed.

### Concentration stability of the *R* and *S*-epimers of ergot alkaloids

Ergot alkaloid concentrations, including the* R* and *S*-epimers, are not stable under various conditions. Multiple variables may affect the concentrations including solvent, pH, light, and temperature (Komarova and Tolkachev 2001; Hafner et al. 2008; Krska et al. 2008a; Lea et al. 2014; Coufal-Majewski et al. 2017; Schummer et al. 2020). The *R*-epimer can convert, or epimerize, to the *S*-epimer in the “presences of alkalis” (Komarova and Tolkachev 2001, p. 506), under aprotic (e.g., acetonitrile, acetone, chloroform) and protic solvents (e.g., methanol or water:methanol mix) at room temperature over days, at 37 °C in various matrices over hours (Smith and Shappell 2002), and during the extraction process of cereals (Krska et al. 2008a). The *S*-epimer may convert to the *R*-epimer in organic solvent, water, or acidic solutions (Komarova and Tolkachev 2001).

Optimal storage conditions for pure standards of *R* and *S*-epimers of ergot alkaloids should be below −20 °C, in non-protic solvents (Hafner et al. 2008; Krska and Crews 2008; Crews 2015), and in amber vials (Smith and Shappell 2002), for accurate concentration assessments of the *R* and *S*-epimers. Further, analytical run time should be minimized to also avoid epimerization throughout the analysis (Diana Di Mavungu et al. 2012). An extraction solvent with pH 8.5 demonstrated optimal extraction of both *R* and* S*-epimers while recognizing that higher pH increase epimerization. Further, a dry down temperature of 40 °C had less epimerization that 60 °C. Extraction procedures with shorten sample preparation and instrument conditions, such as autosampler temperature, help reduce epimerization within analytical methods. The concentration stability and epimerization of the *R* and *S*-epimers may depend on the specific ergot alkaloid assessed (Schummer et al. 2020).

Ergot alkaloids have varying *pK*_a_ values which may contribute to the extent of epimerization (Schummer et al. 2020). The *pK*_a_ values of the *R*-epimers vary from 5.5 (ergocristine) to 6.0 (ergometrine) and the *S*-epimers vary from 4.8 (ergocorninine) to 6.2 (ergometrinine) (Krska et al. 2008b). In addition, steric hindrance may also contribute to the extent of epimerization for specific ergot alkaloids (Schummer et al. 2020). The concentration of ergocristinine has demonstrated near-complete stability at physiological conditions with greater than 90% of the initial dose of ergocristinine remaining following incubation in cell culture media (Mulac et al. 2012). According to quantum calculations, an *S*-epimer configuration was slightly preferred for ergocornine/inine with 60% of the *S*-epimer present at equilibrium, whereas alpha-ergocryptine/inine was balanced (Andrae et al. 2014). However, in solvent mixtures, the *S*-epimers, alpha-ergocryptinine and ergocorninine, were the preferred configuration with *K* values greater than 1 ranging from 1.06 to 2.83, depending on the solvent mixture and alkaloid assessed. The *K* values greater than 1 represent a shift in the equilibrium to the *S*-epimer. Ergovaline converted to ergovalinine at various rates depending on the matrix and pH assessed at 37 °C (Smith and Shappell 2002). For example, in water, acetonitrile, and a solution of 0.1 M PO_4_ pH 3, equilibrium was not reached within 72 h. However, 0.1 M PO_4_ pH 7.5 reached equilibrium within 75 min. In addition, equilibrium was not reached until approximately 11 h in 9.1% fetal bovine serum in water with pH 7.5. Epimerization of the *R* and *S*-epimers depends on multiple factors, one of which is the matrix of ergot contamination.

The *R* and *S*-epimer configurations may behave differently in certain matrices. In raw material contaminated with ergot, the *S*-epimer concentration increased when stored for an extended period or improper storage conditions for a shorter period (Krska et al. 2008a; Tittlemier et al. 2015). In barley extracts, there was a 10% increase in the *S*-epimer concentrations for ergocristinine, ergocryptinine, and ergocorninine when stored at 4 °C over 2 weeks (Krska et al. 2008a). However, in rye extracts under the same conditions, greater than 50% increase in ergocorninine was observed and a high increase in ergocristinine and ergocryptinine concentrations. The sum of the *S*-epimer concentrations have been 36% in grain samples stored for years, and 20% stored for months (Tittlemier et al. 2015).

In biological matrices, the epimer configurations may also vary. In cell medium, equilibrium between *R* and *S*-epimers was observed between 8 and 24 h, depending on the ergot alkaloid (Mulac and Humpf 2011), with uptake and accumulation of ergocristinine within the cells. Ergovaline and ergovalinine crossed human intestinal cells at the same rate; however, epimer ratios in the cells differed as a resulted of decreased ergovaline concentrations (Shappell and Smith 2005), which could be associated with epimerization of the *R* to the *S*-epimer. Similarly, as mentioned above, in media with varying pH’s and buffers at physiological temperature (37 °C), there was conversion of an *R*-epimer, ergovaline, to the *S*-epimer, ergovalinine, which depending on the media used, did not reach epimerization for hours (Smith and Shappell 2002). The concentration of each epimer was not equal once equilibrium was reached in certain media. Epimerization may be induced in the bile and pancreatin (Merkel et al. 2012) and may occur from stomach to large intestine (Dänicke 2016); however, it is also dependent on the ergot alkaloid assessed. Using an in vitro digestion model, ergocorninine, alpha/beta ergocryptinine, and ergocristinine, which are all *S*-epimers, demonstrated an increase in concentrations compared to their corresponding *R*-epimers (Merkel et al. 2012). Conversely, ergotamine and ergosine (*R*-epimers) demonstrated increased concentrations. Epimerization appeared to occur in the duodenal juice section of the method. Very few studies, if any, have described the kinetics and rate of equilibrium of the *S*-epimers to the *R*-epimers in various matrices and biological fluids. From the above studies, it appears that the *S*-epimers may be the favored configuration in the equilibrium, depending on the ergot alkaloid assessed. The conversion of the *R*-epimer to the *S*-epimer may be more energetically favored, and the steric hindrance of the *S*-epimers may limited the epimerization of the *S*-epimers to the *R*-epimers (Andrae et al. 2014; Schummer et al. 2020). Further, the rate of conversion of the *R*-epimer to the *S*-epimers may take hours to days, depending on the experimental conditions assessed. The stability of ergot alkaloids in various matrices may determine the concentration and proportions of each configuration.

### Proportions of ergot epimers

Each ergot sclerotia may contain a variable concentration of ergot alkaloids (Carbonell-Rozas et al. 2021). The total ergot alkaloid concentrations can differ from 0.01 to 1.04% in a single ergot body (Ruhland and Tischler 2008). The six common ergot alkaloids have different percentages within an ergot body depending on type of crop, fungi, individual ergot bodies, year, and geographical location (Grusie et al. 2017; Kodisch et al. 2020). Since the concentrations of ergot alkaloids can vary extensively, certain ergot alkaloids may be more important to assess in specific regions worldwide.

The proportions of ergot epimers differ globally. In Canada, ergocristine and ergocristinine are the most prevalent epimers (Coufal-Majewski et al. 2017; McKinnon et al. 2018; Cherewyk et al. 2023b). Ergocristine and ergocristinine constituted 35 and 17%, respectively, in Canadian wheat samples (Cherewyk et al. 2023b), with the total sum of the* S*-epimers constituting 35% (Cherewyk et al. 2022a). Ergocristinine had the highest concentration of 29–35% of the total ergot alkaloid concentrations in various matrices (Tittlemier et al. 2019). Similarly, ergocristinine had the highest mean concentration of 16.15 µg/kg and 26.75 µg/kg, compared to the other six common *R* and *S*-epimers, in swine and dairy feed samples, respectively (Poapolathep et al. 2021). In Algeria, ergometrine had the highest mean concentration of 13.5 µg/kg and 33.1 µg/kg out of the 12 common epimers analyzed in barley and wheat (Carbonell-Rozas et al. 2021), and was the most frequently detected epimer in Belgian baby food products (Huybrechts et al. 2021). In Slovenia, ergometrine/inine (17% and 15.3%, respectively) and ergosine/inine (13% and 11.5%, respectively) were the most abundant in various matrices (Babič et al. 2020). Ergocristinine (0.11%) and ergotaminine (17%) had the higher concentrations than their corresponding *R*-epimers (0.02 and 2%, respectively) in rye samples from Germany (Kodisch et al. 2020). Ergotamine had the highest concentration (1411 mg/kg) and ergotaminine had the fifth highest (196 mg/kg) concentrations out of 10 analyzed ergot epimers in rye samples (Blaney et al. 2009). Ergotamine also had the highest mean concentration (554 µg/kg) and ergotaminine (71 µg/kg) out of 12 the common epimers analyzed in barley grain (Drakopoulos et al. 2021). It is important to quantify all six common ergot alkaloids since their concentrations vary. In addition, the concentrations of the* S*-epimers of the common ergot alkaloids are found in high concentrations and proportions worldwide. The *S*-epimer concentrations are commonly found at higher concentrations than the *R*-epimers (Arroyo-Manzanares et al. 2017). The high concentrations of the *S*-epimers and their potential bioactivity encourage inclusion of the *S*-epimers in food and feed safety guidelines globally.

### Ergot regulations

The six common ergot alkaloids in all cereals should be monitored (Crews 2015; Schummer et al. 2018; Tittlemier et al. 2019). Ergot alkaloid intoxication is still an issue in developing countries (Maruo et al. 2018; Debegnach et al. 2019; Wielogorska et al. 2019; Agriopoulou 2021), and remains a concern for livestock (Schummer et al. 2020). Specific regulations have been set for the contamination of mycotoxins within grain. For ergot-contaminated grain, visual inspection of the harvested grain is conducted (Walkowiak et al. 2022). Ergot sclerotia are counted and a percent of weight of sclerotia per weight of grain is assessed. Similarly, depending on the level of ergot contamination, the grain is graded to determine how the grain will be processed or utilized. Currently, 0.05% and 0.02% ergot sclerotia in wheat and durum, respectively, are the maximum values set for human food consumption in Canada (Tittlemier et al. 2019). Maximum sclerotia concentrations of 0.5 g/kg and 5 g/kg in wheat and durum, respectively, have been set by Codex Alimentarius for human consumption (Maruo et al. 2018).

The correlation between ergot sclerotia quantity and the concentration of ergot alkaloids is poor (Babič et al. 2020), especially at low ergot alkaloid concentrations (Grusie et al. 2017; Kodisch et al. 2020). Furthermore, some studies assessing the relationship do not quantify all six common ergot alkaloids or the *S*-epimers (Tittlemier et al. 2015; Grusie et al. 2017). Since ergot alkaloid concentrations cannot be reliably predicted based on the amount of ergot sclerotia presence, an analytical method should be used to ensure that ergot alkaloid concentrations meet safety regulations (Coufal-Majewski et al. 2016; Kodisch et al. 2020). Recommendations for the regulations of ergot-contaminated grain should be based on ergot alkaloid concentrations, and not on the presence of ergot sclerotia alone (EFSA 2012; McKinnon et al. 2018).

New regulations for ergot alkaloid concentrations in food stuff include both the *R* and *S*-epimers (European Commission 2021). The lowest concentration within the regulatory recommendations is based on vulnerable populations such as infants and children at 20-µg/kg total ergot alkaloids. Studies from multiple countries reported mean ergot alkaloid concentrations in food stuff that were below or close to the regulatory recommendation (Veršilovskis et al. 2020; Carbonell-Rozas et al. 2021; Huybrechts et al. 2021). However, certain samples had ergot alkaloid concentrations that exceeded the regulatory recommendation; therefore, vulnerable groups may be at risk. For humans, the European Food Safety Authority (EFSA) reported a total daily intake (TDI) of ergot alkaloids of 0.6-µg/kg body weight per day total ergot alkaloids, and an acute reference dose of 1-µg/kg body weight (EFSA 2012; Debegnach et al. 2019; Malir et al. 2023). The total ergot alkaloids refer to the sum of the six common* R* and *S*-epimers (Carbonell-Rozas et al. 2022). The TDI is the estimated concentration that can be consumed over a lifetime without risk to health, and an acute reference dose is the maximum concentration that can be consumed in a day without risk (Liu and Chen 2003). Guidelines may vary from country to country related to variable consumption of contaminated food products, and the intended population consuming the products. Not only should the *S*-epimers of ergot alkaloids be included in food safety recommendations, but they should be included in feed safety recommendations as well.

Harvested grain and screened grain contaminated with ergot may be used for animal feed (Coufal-Majewski et al. 2016). The European Commission 2002 set a limit of 1000 mg/kg of rye ergot (*Claviceps purpurea*, sclerotia) in feedstuff without ground cereal, and was not changed in the updated European Commission 2011. The US limit for ergot in cereals is 0.3% ergot sclerotia and Australia and New Zealand set limits of 0.05% (Scott 2009). In Canada currently, limits are set for the concentration of ergot alkaloids in animal feed; however, only the *R*-epimers of ergot alkaloids are considered (CFIA, 2017). In Uruguay, there is a limit of 450-μg/kg total alkaloids in feed; however, there is a zero tolerance for vulnerable populations (Scott 2009). A concentration of 200–250-µg/kg ergot alkaloids has been recommended for higher risk livestock such as pregnant or lactating animals (Coufal-Majewski et al. 2016; Lea and Smith 2021). In a case study, multiple beef claves had died with concentrations of total ergot alkaloids (only *R*-epimers reported) in the diet reaching 495 µg/kg (Leuschen et al. 2014). The concentration of both *R* and *S*-epimers should be accounted for in feed for livestock as reported by government and industry of the protection of animal health.

Many countries worldwide have reported a high percent of ergot-contaminated feed samples. Studies have demonstrated that the average concentration of ergot alkaloids in feed were below safety standards; however, specific samples had ergot alkaloid concentrations that were higher than the recommended safety standards in various matrices (Schummer et al. 2018; Babič et al. 2020; Kodisch et al. 2020; Arroyo-Manzanares et al. 2021; Poapolathep et al. 2021). Concentrations lower than safety standard guidelines, such as 100–200-µg/kg total ergot alkaloids, have produced adverse effects to livestock (Evans 2011; Craig et al. 2015; Miskimins et al. 2015; Coufal-Majewski et al. 2016; Cowan et al. 2018; Arroyo-Manzanares et al. 2021).

Current regulatory standards may not provide sufficient protection against ergot alkaloids (Maruo et al. 2018); therefore, ongoing studies are needed to re-evaluate these standards until adequacy is met (Debegnach et al. 2019) and include the *S*-epimers in feed standards for the protection of animal health. As the analytical detection of *R* and *S*-epimers is more fully understood, greater consistency of guidelines will occur. The use of a marker compound would not be useful for ergot alkaloid analysis based on varying ergot alkaloid compositions worldwide (Klotz et al. 2016; Tittlemier et al. 2019; Drakopoulos et al. 2021), and the toxicities of the individual ergot alkaloids are not well understood (Arroyo-Manzanares et al. 2017). In addition, certain alkaloids behave differently in terms of their concentrations when exposed to various external factors (Roberts et al. 2014; Schummer et al. 2020); therefore, the analysis of one ergot alkaloid as a marker may not reflect the concentration of other ergot alkaloids. Initially, the inclusion of the *S*-epimers in monitoring guidelines was associated with the potential for this configuration to convert back to the *R*-epimers (EFSA 2012). However, the *S*-epimers should also be included in the guidelines based on the potential bioactivity of the *S*-epimers which may lead to adverse effects (Mulac et al. 2012; Cherewyk et al. 2020, 2022c), and high concentrations in natural contaminated matrices (Tittlemier et al. 2019; Cherewyk et al. 2022a). In addition, the extent of the *S*-epimer bioactivity and stability in terms of their concentrations needs to be further researched to gain a greater understanding of their effects. The full impact on existing ergot guidelines and food and feed safety remains unknown.

## Conclusions

Globally, ergot alkaloid contamination is an issue in food and feed and can effect the health and well-being of humans and animals. Historical and current studies on the C-8-*S*-isomers have been collected to demonstrate and highlight the progression of C-8-*S*-isomer research and compare it to the C-8-*R*-isomer. The biological and analytical aspects of the C-8-*S*-isomers are important to consider because of the potential effects to the health of humans and animals.

This review reveals the importance of the inclusion of the C-8-*S*-isomer in future research and may aid in assessing the C-8-*S*-isomers. Future research on the mechanisms and biological effects of the C-8-*S*-isomers is warranted. It is important to quantify the C-8-*S*-isomers in contaminated matrices since the concentration may be high. Once there is a greater understanding of the C-8-*S*-isomers, acceptance of the C-8-*S*-isomers into the regulatory recommendations for food and feed worldwide for the protection of human and animal health may occur.

## Data Availability

Data sharing not applicable to this article as no datasets were generated or analysed during the current study.
